# Rigid sellar floor reconstruction in transsphenoidal surgery: prevention of cerebrospinal fluid leakage attributable to the water-hammer effect

**DOI:** 10.1007/s10143-026-04408-5

**Published:** 2026-07-17

**Authors:** Kosaku Amano, Yuichi Oda, Takakazu Kawamata

**Affiliations:** Department of Neurosurgery, Tokyo Women’s Medical University, 8-1 Kawada-cho, Shinjuku-ku, Tokyo, 162-8666 Japan

**Keywords:** Transsphenoidal surgery, Sellar floor reconstruction, Cerebrospinal fluid leakage, Rigid buttress, Water-hammer effect, Autologous bone graft

## Abstract

Postoperative cerebrospinal fluid (CSF) leakage remains one of the most challenging complications of transsphenoidal surgery (TSS). Numerous reconstruction techniques have been proposed, but none has been established as the gold standard. To address this issue, we standardized rigid sellar floor reconstruction using a hard buttress, based on the concept of counteracting the water-hammer effect of pulsatile CSF pressure. We retrospectively analyzed 1,168 consecutive TSS procedures for sellar lesions performed by a single surgeon between October 2004 and March 2024. Routine rigid reconstruction was implemented from January 2009 (Group B, *n* = 856) and compared with an earlier cohort without routine buttressing (Group A, *n* = 312). Reconstruction materials included autologous bone grafts, calcium phosphate cement, titanium mesh, and resorbable plates. Rigid reconstruction was performed in 885 cases, most commonly with autologous bone (*n* = 781), which proved most reliable. Calcium phosphate cement occasionally caused inflammatory change or graft dislodgement, and titanium mesh complicated reoperations. Postoperative CSF leakage requiring reoperation was significantly reduced from 2.88% in Group A to 0.47% in Group B (*P* = 0.002), despite a higher intraoperative leak rate in Group B (76.5% vs. 54.2%, *P* < 0.0001), particularly grade 3 leaks (30.4% vs. 11.2%, *P* < 0.0001). All postoperative leaks in Group B were attributable to inadequate buttressing. Rigid sellar floor reconstruction is a fundamental strategy—together with dural suturing and vascularized mucosal flaps—for safer and more extensive resections in modern TSS, supported by a physiological rationale based on the water-hammer effect.

## Introduction

Transsphenoidal surgery (TSS) is a well-established approach for the removal of pituitary and parasellar tumors. Nevertheless, intraoperative cerebrospinal fluid (CSF) leakage, which occurs more frequently with aggressive tumor removal, represents a major intraoperative challenge and, consequently, postoperative CSF leakage remains one of the most critical complications [1–3]. Numerous preventive techniques have been described, including autologous grafts, alloplastic materials, dural suturing, rigid reconstruction, and vascularized mucosal flaps; yet no single method has proven completely effective in eliminating leakage [4–26]. These observations highlight the need for a rational, mechanism-informed strategy that integrates complementary methods rather than relying on any single technique. Such an integrated, mechanism-based approach may also shorten the learning curve in TSS, as described by Ciric et al. [1], thereby contributing to a reduction in this complication.

To minimize postoperative CSF leakage, a standardized multilayer closure strategy has been adopted in TSS, consisting of dural suturing [7, 10], rigid sellar floor reconstruction, and a sphenoid sinus mucosal flap [23], with the aim of restoring the native barrier anatomy as closely as possible. While the effectiveness of dural suturing and vascularized mucosal flaps has been reported previously, the present study focuses on rigid sellar floor reconstruction using a hard buttress. In addition, the mechanistic basis of postoperative CSF leakage is examined, with particular emphasis on the water-hammer effect.

## Materials and methods

Between October 2004 and March 2024, a total of 1,168 consecutive TSS procedures were analyzed. Inclusion criteria comprised all TSS procedures requiring fenestration of the sella turcica, including both standard sellar and extended transsphenoidal approaches, performed by the first author during the study period. Procedures not involving sellar floor fenestration were excluded. The cohort included 488 men and 680 women, with an age range of 3–86 years (mean, 49.9 years; median, 54.0 years). The underlying pathologies or indication included 741 pituitary neuroendocrine tumors (PitNETs), 235 Rathke’s cleft cysts, 50 craniopharyngiomas, 30 inflammatory lesions, 21 xanthogranulomas, 17 CSF leakages, 13 chordomas, 10 meningiomas, 9 metastatic tumors, 5 arachnoid cysts, and 38 other lesions (Table 1). Postoperative CSF leakage was defined as clinically evident leakage requiring surgical re-exploration. In accordance with our institutional strategy, suspected postoperative leakage was not managed conservatively with lumbar drainage or prolonged bed rest, but was instead promptly treated with surgical repair. All patients were followed clinically for the detection of postoperative CSF leakage during the postoperative period and subsequent outpatient follow-up. All postoperative CSF leakage events were observed within the first postoperative month, and the minimum follow-up duration for all patients exceeded one year.

**Table 1:** Characteristic of 1168 Trans–sphenoidal Surgery

	Overall	A	B
Men: Women	488: 680	126: 186	362: 554
Age (years–old) range	3–86	5–85	3–86
Mean, median	49.9, 54.0	44.9, 45.0	51.7, 53.0
Pathology or indication	n (%)	n (%)	n (%)
Pituitary neuro–endocrine tumor	741 (63.4)	212 (68.0)	529 (61.8)
Rathke’s cleft cyst	235 (20.1)	56 (18.0)	179 (20.9)
Craniopharyngioma	50 (4.3)	8 (2.6)	42 (4.9)
Inflammatory disease	30 (2.6)	3 (1.0)	27 (3.2)
Xanthogranuloma	21 (1.8)	5 (1.6)	16 (1.9)
CSF leakage*	17 (1.5)	11 (3.5)	6 (0.7)
Chordoma	14 (1.2)	5 (1.6)	9 (1.1)
Meningioma	10 (1.1)	1 (0.3)	9 (1.1)
Metastatic tumor	7 (0.9)	0 ())	7 (0.8)
Arachronoid cyst	5 (0.4)	2 (0.6)	3 (0.4)
Others	38 (3.3)	9 (2.9)	29 (3.4)
Total	1168	312	856

*This group included two reoperations, one proclamation under cabergoline therapy, and one case previously operated at another institution.

### Rationale for rigid sellar floor reconstruction

Based on the principle that the sellar floor, being originally osseous, should be reconstructed using bone or an equivalent rigid material, rigid reconstruction of the sellar floor was initiated in February 2007 with the use of calcium phosphate cement. The utility of rigid reconstruction was subsequently recognized, as CSF leakage was absent when the cement adequately solidified, whereas leakage occurred when sufficient hardening was not achieved. The utility of rigid reconstruction was subsequently recognized, as CSF leakage was absent when the cement adequately solidified, whereas leakage occurred when sufficient hardening was not achieved. Following these observations, additional reconstructive materials were introduced in a stepwise manner: autologous bone grafts harvested from the nasal cavity—previously discarded tissue—from October 2008 onward; titanium mesh plates from November 2008 onward; and resorbable fixation plates (LactoSorb^®^, Biomet Microfixation, Jacksonville, FL, USA) from January 2009 onward. From January 2009, rigid sellar floor reconstruction was routinely performed in virtually all cases, reflecting its recognized necessity (Table 2). This approach was applied regardless of tumor type, surgical approach (standard sellar or extended), or the presence or apparent absence of intraoperative CSF leakage, as subtle CSF fistulas or air ingress into the intracranial space were occasionally observed even in cases without overt leakage.

**Table 2:** Materials Used for Rights Reconstruction

	Duration of clinical use	Overall	A	B
Calcium phospate cement	Feb. 2007–Mar.2008	60	60	0
Autologous bone graft from the nasal cavity	Oct. 2008–	781	8	773
Titanium mesh plate	Nov. 2008–Feb. 2012	34	1	33
Resorbable fixation plate	Jan. 2009–	885	0	10
Total				

## Characteristics and methods of use for each material

### Calcium phosphate cement (CPC)

**Fig. 1:**
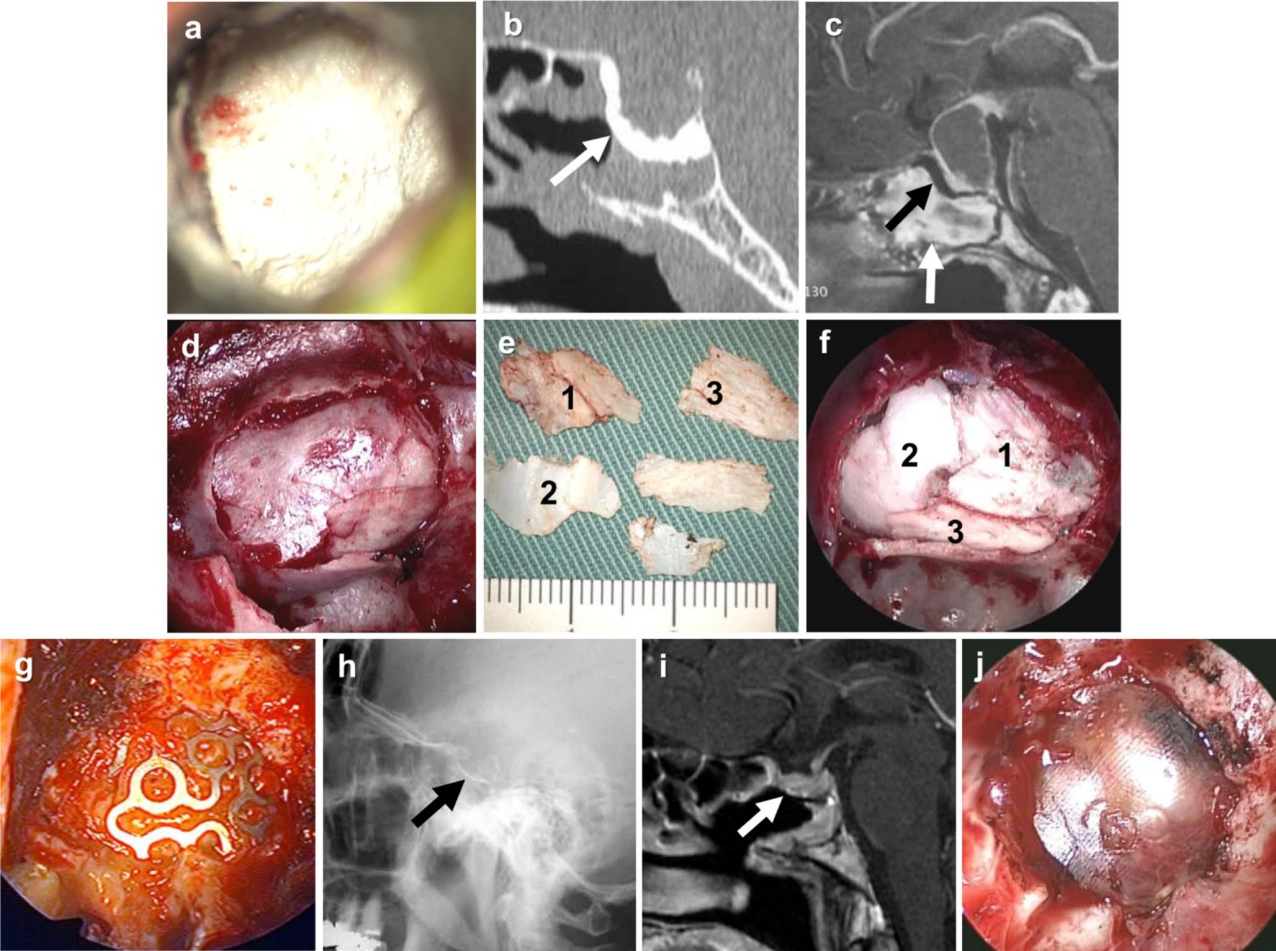
(**a**) Intraoperative view showing rigid sellar floor reconstruction using calcium phosphate cement (CPC) paste. (**b**) Sagittal bone-window CT scan demonstrating CPC paste reconstruction of the sellar floor (white arrow). (**c**) Contrast-enhanced sagittal T1-weighted MRI showing CPC paste reconstruction (black arrow) and inflammatory changes within the sphenoid sinus (white arrow). (**d**) Intraoperative view of sellar floor reconstruction using a single nasal septal bone fragment. Gelatin sponge was placed in the epidural space, and the bone fragment was inserted extradurally beneath the bony edge of the sellar window. (**e**) Harvested nasal septal bone fragments prepared for reconstruction. (**f**) Sellar floor reconstruction using three bone fragments, each corresponding to the numbered fragments shown in panel e. (**g**) Intraoperative view of sellar floor reconstruction using a titanium mesh plate. (**h**) Lateral radiograph demonstrating the titanium mesh plate (black arrow). (**i**) Contrast-enhanced sagittal T1-weighted MRI demonstrating the titanium mesh plate (white arrow). (**j**) Intraoperative view of sellar floor reconstruction using a resorbable fixation plate (Lactosorb^®^)

Clay-like CPC was applied over the bony defect and its margins in a putty-like manner to recreate the sellar floor. Since the freshly mixed paste is initially too soft, it was best applied once partial hardening had begun. As moisture interferes with proper solidification, the surgical field was carefully dried with cotton pledgets and suction before application, after which the CPC was molded to shape the sellar floor (Fig. 1a–c).

## Autologous bone graft from the nasal cavity

**Fig. 2:**
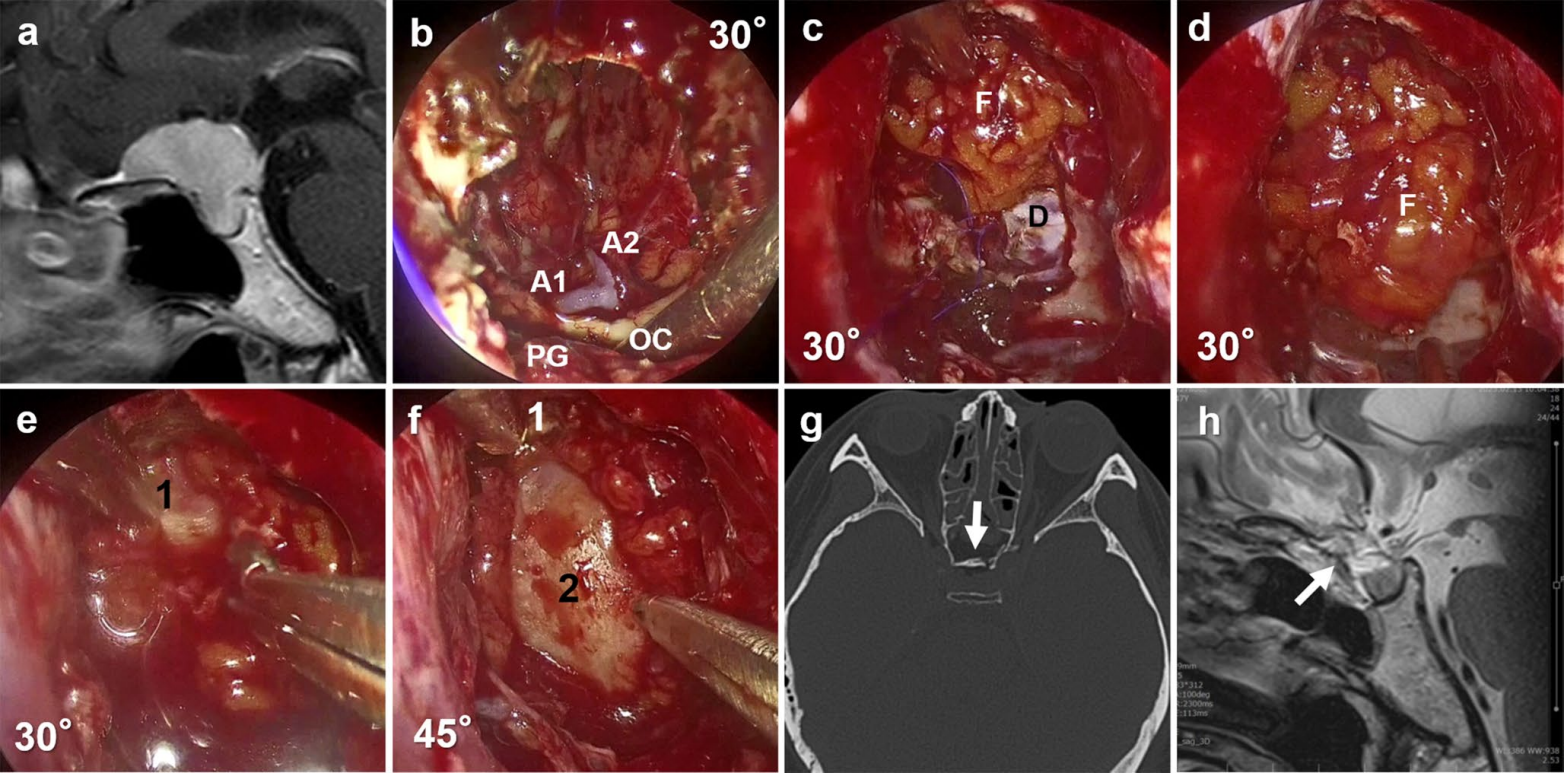
(**a**) Preoperative contrast-enhanced sagittal T1-weighted MRI of a 49-year-old woman with a tuberculum sellae meningioma. (**b**) Endoscopic view after tumor removal using a 30° endoscope (A1 = A1 segment of the anterior cerebral artery; A2 = A2 segment of the anterior cerebral artery; OC = optic chiasm; PG = pituitary gland). (**c**) Dural closure with autologous fat using a 30° endoscope (D = dura; F = fat). (**d**) Autologous fat graft covering the dural closure using a 30° endoscope (F = fat). (**e**) Insertion of bone fragment 1 in a horizontal orientation along the anterior edge of the bony window using a 30° endoscope. (**f**) Insertion of bone fragment 2 using a 45° endoscope, with bone fragment 2 hooked between the lower margin of bone fragment 1 and the inferior edge of the bony window. (**g**) Postoperative axial bone-window CT scan and (**h**) sagittal T2-weighted MRI demonstrating the wedged autologous bone fragment (white arrow)

Bone fragments were harvested from the nasal septum, the anterior wall of the vomer, or the intersinus septum of the sphenoid sinus. The purpose was to suppress dural pulsation; therefore, it was not always necessary to cover the entire bony defect, but the graft had to be firmly wedged into the extradural space and beneath the bony edges of the defect to ensure stability. After placement, stability was confirmed by gently tapping with a suction tip. Ideally, the entire defect was covered, and when necessary, multiple pieces were fitted together like a puzzle(Fig. 1d-f). In cases where the harvested bone was insufficient, an elongated bone fragment was stabilized by inserting it so that both ends were hooked into the bony edge, thereby achieving fixation analogous to a clamp Fig. 2f.

Any remaining harvested bone was always repositioned into the submucosa of the nasal septum. Care was taken not to injure the sphenopalatine artery during replacement. Cartilage was not harvested to avoid alteration of nasal shape.

## Titanium mesh plate

The same technique as for autologous bone was applied. The plate was trimmed with nippers to be slightly larger than the bony defect and rounded to match the contour of the sellar floor. No artifacts were observed on postoperative MRI(Fig. 1g-i).

### Resorbable fixation plate (RFP)

The same technique as for autologous bone was applied. An advantage of RFP is that it softens at approximately 60 °C, becoming moldable. However, although it remains pliable while immersed in warm water, it tends to cool and harden before reaching the sellar floor. To avoid this, the plate was wrapped in a warm, saline-soaked pledget and delivered together with it. If further adjustment was needed, it was reheated intraoperatively with a warm pledge, allowing reshaping(Fig. 1j). RFP was absorbed approximately one year postoperatively.

## Study groups and intraoperative cerebrospinal fluid leakage assessment

Patients were divided into two groups according to whether rigid reconstruction had been routinely implemented: Group A (October 2004–December 2008, *n* = 312) before its adoption, and Group B (January 2009–March 2024, *n* = 856) after its adoption.

Intraoperative CSF leakage was assessed and graded according to the Esposito classification [26]. For cases treated from 2009 onward, grading was assigned intraoperatively by the operating surgeon and recorded prospectively in the operative records. For cases treated before 2009, grading was retrospectively assigned based on the operative records. Lower-grade leaks correspond to low-flow leakage, whereas higher-grade leaks, including grade 3, correspond to high-flow leakage, as commonly defined in the literature (Table 3).

**Table 3:** Incidence of Intraoperative Cerebrospinal Fluid (CSF) Leakage and Postoperative CSF Leakage Requiring Reoperation Before and After the Introduction of Routine Rigid Reconstruction

Group		Intraoperative CSF leakage					Postoperative CSF lea
			0 (n=344)	1 (n=237)	2 (n=292)	3 (n=295)	
A	Oct. 2004–Dec. 2008 (n=312)	169	143	50	84	35	9
	%	54.17	45.83	16.03	26.92	11.22	2.88
B	Jan. 2009–Mar. 2024 (n=856)	655	201	187	208	260	4
	%	76.52	23.48	21.84	24.29	30.37	0.47
	*P* value	<.0001				<.0001	.002

## Ethical considerations and statistical analysis

This study was approved by the Ethics Committee of our institution (approval number: 2021-0063) and conducted in accordance with the Declaration of Helsinki and the STROBE guidelines. Given the retrospective nature of the study, the requirement for written informed consent was waived by the Ethics Committee, and an opt-out method was employed for the academic use of clinical data. Proportions are reported with two-sided 95% confidence intervals (CIs) using the Wilson score method. Risk ratios (RRs) are presented with 95% CIs calculated on the log scale. Two-sided *p* < 0.05 was considered statistically significant, with χ² or Fisher’s exact tests used as appropriate.

## Results

### Patient characteristics

A total of 1,168 consecutive TSS procedures meeting the predefined inclusion criteria, as described in the Methods section, were analyzed. The distribution of underlying pathology or surgical indication is summarized in Table 1. PitNETs were the most common (63.4%), followed by Rathke’s cleft cysts (20.1%) and craniopharyngiomas (4.3%). Craniopharyngiomas and meningiomas—both associated with high risk of intraoperative CSF leakage—were more frequent in Group B (6.0%) than in Group A (2.9%) (*p* = 0.036). Seventeen cases (1.5%) were categorized as “CSF leakage” cases, including two reoperations, one prolactinoma under cabergoline therapy, and one case initially operated at another institution.

### Materials used for rigid reconstruction

Rigid reconstruction was performed in 885 cases (69 in group A and 816 in group B), most commonly with autologous bone grafts harvested from the nasal cavity (*n* = 781; October 2008 onward). Calcium phosphate cement was used in 60 cases (February 2007–March 2008), titanium mesh plates in 34 cases (November 2008–February 2012), and resorbable fixation plates in 10 cases (January 2009 onward). Among the 856 cases in Group B, rigid reconstruction was not performed in 40 cases in which the sellar floor was destroyed and lacked a bony edge to secure a hard buttress, or when only a limited biopsy was performed (Table 2).

### Incidence of CSF leakage before and after routine rigid reconstruction

The incidence of postoperative CSF leakage requiring reoperation decreased from 2.88% (95% CI, 1.52–5.39; 9/312) in Group A to 0.47% (95% CI, 0.18–1.20; 4/856) in Group B (RR, 0.16; 95% CI, 0.05–0.52; *P* = 0.002). Conversely, the incidence of intraoperative CSF leakage was higher in Group B than in Group A: 76.52% (95% CI, 73.56–79.24; 655/856) vs. 54.17% (95% CI, 48.62–59.61; 169/312) (RR, 1.41; 95% CI, 1.27–1.57; *P* < 0.0001). Specifically, grade 3 intraoperative leakage occurred more frequently in Group B: 30.37% (95% CI, 27.39–33.54; 260/856) vs. 11.22% (95% CI, 8.18–15.20; 35/312) (RR, 2.71; 95% CI, 1.95–3.76; *P* < 0.0001) (Table 3).

### Complications associated with rigid reconstruction

Among the 60 cases reconstructed with calcium phosphate cement, complications included abscess formation (Fig. 1c) occurred in 2 (3.3%) cases (both Rathke’s cleft cysts), sinusitis in 15 (25%), and graft dislodgement in 5 (8.3%). Postoperative CSF leakage requiring reoperation in Group A (*n* = 312) occurred in 9 cases: 4 GH-secreting PitNETs, 4 non-functioning PitNETs, and 1 ACTH-secreting PitNET. Calcium phosphate cement was used for rigid reconstruction in 2 of these 9 cases. In Group B (*n* = 856), postoperative CSF leakage requiring reoperation occurred in 4 cases reconstructed with autologous bone: 2 non-functioning PitNETs, 1 GH-secreting PitNET, and 1 Rathke’s cleft cyst. In one case, the initially inserted anterior bone fragment was displaced superiorly by a subsequently inserted posterior fragment, perforating the arachnoid. In the remaining three cases, the grafts were not firmly anchored to the bony edge and became displaced. In the titanium plate group, one of 34 cases required reoperation; removal was difficult because the plate had become embedded in bone, necessitating drilling. No complications related to the use of resorbable fixation plates as a hard buttress were observed.

## Discussion

Postoperative CSF leakage remains a critical complication of TSS. Over the years, numerous preventive methods have been proposed, but no single technique reliably eliminates leakage completely; optimal outcomes require a combination of strategies.

In craniotomy, CSF leakage rarely occurs because the dura is sutured watertight, the bone flap is replaced, and the scalp closed in layers. If the same principles could be applied to TSS, CSF leakage would likewise be expected to be rare. However, the anatomical constraints of TSS—namely the deep and narrow surgical corridor—make watertight dural closure, bony replacement, and multilayered vascularized coverage technically challenging. The value of dural suturing in TSS and vascularized mucosal flaps—particularly the minimally invasive sphenoid sinus mucosal flap— has been shown in prior studies [7, 10, 23]. The present study focuses on the role, importance, and mechanisms of rigid sellar floor reconstruction as the remaining key element for preventing CSF leakage.

### Mechanism of CSF leakage and water-hammer effect

The following discussion is intended to present a conceptual hypothesis to explain the observed reduction in postoperative CSF leakage, rather than a directly measured physiological mechanism.

In craniotomy, restoring the bone flap serves three main purposes: (1) protection of the brain from external trauma, (2) cosmetic benefit, and (3) protection of the brain from atmospheric pressure. In TSS, the first two functions are largely irrelevant, because the sella is not exposed externally and cosmetic concerns do not arise; however, the third function is highly pertinent. Indeed, Matsuda et al. reported cases of tension pneumosella after TSS without rigid reconstruction [27]. Beyond these three purposes, an additional physiological rationale likely exists for the sellar bone—particularly since in infancy the conchal type sella persists as a thick bony structure without sphenoid sinus pneumatization. This anatomical persistence suggests that the sellar bone is not merely structural but may serve a physiological role. The sellar floor may function as a damping and buffering structure for CSF pulsation.

In the fields of hydraulics and engineering, there have been numerous reports on the water-hammer effect (WHE) [28, 29]. The WHE refers to the generation of a pressure surge when a moving fluid is abruptly decelerated, classically observed in pipelines when a valve is suddenly closed. This rapid conversion of kinetic energy into pressure produces a high-magnitude impulse wave.

Anatomically and hydrodynamically, the sella is a region highly susceptible to CSF pulsatile pressure, a vulnerability that underlies phenomena such as empty sella [30]. When a bony defect of the sellar floor is left unreconstructed, CSF pulsatile pressure is concentrated on this anatomically vulnerable region, thereby potentially predisposing it to a water-hammer–like effect. Analogously, repetitive CSF pulsations exert impulse-like forces on the sellar reconstruction, which may compromise the integrity of the repair.

While the CSF pulsatility and related hydrodynamic forces have been discussed in other neurosurgical contexts [31], such as empty sella [30] and syringomyelia [32], the water-hammer effect has not previously been explicitly applied as a mechanistic framework for postoperative CSF leakage after TSS or for rationalizing rigid sellar buttressing. The present study supports the clinical relevance of this concept as a plausible explanatory framework in TSS, thereby addressing a previously unrecognized mechanistic gap.

This interpretation is consistent with studies on intracranial pulsatility, which have shown that increased intracranial pressure reduces craniospinal compliance and amplifies CSF pulse amplitude [33]. Although fat or fascia grafts combined with dural suturing and vascularized flaps may provide temporary watertight closure, delayed CSF leakage can still occur if the reconstructed barrier remains structurally fragile under repeated pulsatile stress [34, 35]. In contrast, rigid buttressing provides mechanical resistance to these pulsatile forces, thereby reducing the risk of delayed CSF leakage.

### Role and drawbacks of lumbar drainage

Lumbar drainage (LD) has long been used to mitigate pulsatile CSF forces. Intraoperative LD reduces arachnoid tension and lowers intraoperative leak rates, and postoperative LD may decrease CSF pressure and facilitate reconstruction healing [36–40]. Clinical studies and technical reports, including a randomized trial and recent large endoscopic endonasal series, have emphasized the importance of tailored reconstruction strategies for reducing postoperative CSF leakage in selected high-risk cases, particularly in extended transsphenoidal procedures [41–44]. However, its routine use with strict bed rest is limited by associated complications, patient distress, prolonged hospitalization, and infection risk, especially problematic in children, where immobilization requires deep sedation or anesthesia. For these reasons, routine LD was not adopted and rigid sellar floor reconstruction was employed as a more reliable alternative. By directly resisting CSF pulsatility, rigid reconstruction provides a physiological effect comparable to LD while avoiding its drawbacks, and plays a crucial role in preventing delayed postoperative CSF leakage.

### Practical aspects, advantages, and limitations of rigid sellar floor reconstruction

In addition to high-flow CSF leaks, risk factors for postoperative CSF leakage after TSS include (1) multiple prior surgeries, (2) radiotherapy, and (3) obesity [45–49]. Factors (1) and (2) render the sellar region structurally fragile, making it unable to withstand CSF pulsatility, whereas obesity contributes through elevation of intracranial pressure. Therefore, performing secure rigid reconstruction in such cases is essential.

Among available materials for rigid reconstruction, autologous bone (AB) is preferred because it avoids foreign-body reactions, is easily harvested during the approach, and can be trimmed and curved for smooth insertion between the dura and the margin of the bony window, where it can be firmly secured. Venous bleeding during insertion is minor and controllable with gelatin sponge or fibrin glue. Furthermore, the rich vascularity of this region allows AB to survive and incorporate; removal during reoperation often requires drilling. Accordingly, AB should be used whenever possible, except in reoperations without remaining bone or in extended TSS requiring a large buttress. Donor sites include the nasal septum, anterior sphenoid wall, and intersinus septum. Harvesting superior septal bone risks olfactory disturbance or saddle nose deformity, but inferior septal bone is often adequate. In patients with prior surgery and septal bone is absent, we routinely reinsert unused fragments beneath the septal mucosa at closure. This technique, adapted from septoplasty in otolaryngology, also prevents septal perforation and preserves bone for reuse in future reoperations.

When AB is insufficient, artificial materials such as titanium or resorbable fixation plates can be useful. The use of calcium phosphate cement was discontinued in March 2008 because of frequent complications, including abscess formation, sinusitis, and graft dislodgement. Titanium plates, being difficult to remove at reoperation and poorly covered by mucosa, should be avoided in patients with reoperation potential. Resorbable fixation plates, although initially too rigid to insert, can be softened with a cotton pledget soaked in warm saline and shaped for use, making them a practical alternative. The material is resorbed within about one year; we place small bony fragments between the plate and the dura as a precaution. Consequently, AB is our preferred material, and resorbable fixation plates are used when the available AB is insufficient (Fig 2).

Rigid reconstruction not only resists CSF pulsatility but also eliminates gaps by compressing epidural fat against the bony margins, thereby achieving a watertight seal, as in the “gasket-seal” technique. In this sense, the “gasket-seal” technique is ideal [50], as it combines rigid buttressing with a watertight closure, similar to the principle of an airtight container lid. Furthermore, when rigid reconstruction securely performed, a less invasive sphenoid sinus mucosal flap is sufficient [23], without the need for a nasoseptal flap, which alters normal anatomical structures. Although some advocate rigid reconstruction only for high-flow leaks such as meningiomas or craniopharyngiomas, routine application is supported. The sellar floor is originally composed of bone, and reconstruction with rigid material restores its native anatomy without alteration. In addition, even in cases without intraoperative CSF leakage, postoperative CT can sometimes demonstrate suprasellar air. For these reasons, rigid sellar floor reconstruction is routinely performed in all cases, regardless of intraoperative cerebrospinal fluid leak status. We acknowledge that excellent results have been reported with simpler closure techniques [51]. Nevertheless, our preferred strategy is rigid sellar floor reconstruction using autologous material, based on the concept of anatomical restoration of the native sellar floor whenever feasible.

The present study highlights the value of rigid reconstruction: postoperative CSF leakage requiring reoperation was significantly reduced after it was standardized (0.47% vs. 2.88%, *p* = 0.002). In contrast, intraoperative CSF leakage was more frequent and severe in Group B than in Group A (overall incidence: 76.5% vs. 54.2%; Esposito grade 3: 30.4% vs. 11.2%; *p* < 0.0001), likely because the proportions of craniopharyngioma and meningioma—both associated with a high risk of intraoperative CSF leakage—were higher in Group B (6.0%) than in Group A (2.9%) (*p* = 0.036; Tables 1 and 3). These findings suggest that advances in leak management, particularly the routine implementation of rigid reconstruction, have enabled broader exposure and safer, more aggressive tumor resections in modern TSS.

Notably, all four cases of postoperative CSF leakage after 2009 were attributable to inadequate buttressing (dislodgement of the inserted bone), underscoring rigid reconstruction as an essential strategy for preventing postoperative leakage. These failures occurred in standard transsphenoidal procedures (three pituitary neuroendocrine tumors and one Rathke’s cleft cyst) and were not associated with extended approaches or lesions typically linked to high-flow cerebrospinal fluid leakage. Importantly, postoperative leakage in these cases was attributable solely to insufficient seating of the hard buttress in the epidural space beneath the bony margins, rather than to intraoperative CSF leak grade or anatomical factors such as third ventricular involvement or suprasellar extension.

### Limitations

A limitation of this study is its single-surgeon, retrospective, before–after design, which may introduce potential temporal confounding related to the learning curve. Advances in visualization, instrumentation, perioperative care, and technical refinements over two decades could also have influenced outcomes. Nevertheless, given that no postoperative CSF leakage occurred in approximately 400 consecutive cases during the initial 5 years and 10 months immediately after rigid reconstruction was standardized, and that four cases of postoperative CSF leakage were observed in the subsequent decade without any apparent temporal clustering, the influence of the learning curve is likely negligible. Given the very low number of postoperative CSF leakage events in Group B, formal multivariable or time-trend analyses would be statistically underpowered and potentially misleading.

Since 1998, dural suturing has been routinely performed, and since 2007, vascularized mucosal flaps have been adopted; both techniques have contributed to a reduction in CSF leakage. However, because dural suturing was performed throughout the entire study period, its effect on postoperative leakage is unlikely to explain the subsequent improvement. During the period after vascularized mucosal flaps were adopted but before routine rigid reconstruction, the rate of postoperative CSF leakage decreased from 4.6% (7/151) to 3.1% (5/162), although the difference was not statistically significant (*p* = 0.56). This modest improvement indicates that the marked reduction to 0.47% was achieved only after routine rigid sellar floor reconstruction had been implemented.

Although CSF pulsatility and intracranial pressure were not directly measured in this study, the proposed water-hammer effect should be interpreted as a physiologically plausible explanatory hypothesis consistent with the observed outcomes. Detailed information on revision surgery, prior radiotherapy, and other potential high-risk subgroups was not available in sufficient detail to allow reliable stratified analyses. Therefore, formal subgroup analyses were not performed to avoid overinterpretation of subgroup effects. Finally, although multivariable adjustment and a formal early-versus-late analysis within Group B were not performed, the consistent results across a large consecutive series underscore the practical value of rigid reconstruction as an integral component of a standardized multilayer closure.

## Conclusions

Rigid sellar floor reconstruction with a hard buttress represents a fundamental strategy in modern TSS. By counteracting the water-hammer effect of CSF pulsatility, it achieves physiological benefits comparable to LD while avoiding its associated complications. In our large consecutive series, routine rigid reconstruction markedly reduced postoperative CSF leakage, even with severe intraoperative leaks, establishing it as a fundamental technique for safer and more effective tumor resections.

## Data Availability

The datasets during and/or analysed during the current study are available from the corresponding author on reasonable request.

## Abbreviations

CSF: cerebrospinal fluid
TSS: transsphenoidal surgery
LD: lumbar drainage
WHE: water–hammer effect
AB: Autologous bone
RR: relative risk
CI: confidence interval
